# State-of-the-art Dashboards on Clinical Indicator Data to Support Reflection on Practice: Scoping Review

**DOI:** 10.2196/32695

**Published:** 2022-02-14

**Authors:** Bernard Bucalon, Tim Shaw, Kerri Brown, Judy Kay

**Affiliations:** 1 Human Centred Technology Cluster School of Computer Science The University of Sydney Darlington Australia; 2 Practice Analytics Digital Health Cooperative Research Centre Sydney Australia; 3 Research in Implementation Science and e-Health Group Faculty of Medicine and Health The University of Sydney Sydney Australia; 4 Professional Practice Directorate The Royal Australasian College of Physicians Sydney Australia

**Keywords:** practice analytics dashboards, data visualization, reflective practice, professional learning, mobile phone

## Abstract

**Background:**

There is an increasing interest in using routinely collected eHealth data to support reflective practice and long-term professional learning. Studies have evaluated the impact of dashboards on clinician decision-making, task completion time, user satisfaction, and adherence to clinical guidelines.

**Objective:**

This scoping review aims to summarize the literature on dashboards based on patient administrative, medical, and surgical data for clinicians to support reflective practice.

**Methods:**

A scoping review was conducted using the Arksey and O’Malley framework. A search was conducted in 5 electronic databases (MEDLINE, Embase, Scopus, ACM Digital Library, and Web of Science) to identify studies that met the inclusion criteria. Study selection and characterization were performed by 2 independent reviewers (BB and CP). One reviewer extracted the data that were analyzed descriptively to map the available evidence.

**Results:**

A total of 18 dashboards from 8 countries were assessed. Purposes for the dashboards were designed for performance improvement (10/18, 56%), to support quality and safety initiatives (6/18, 33%), and management and operations (4/18, 22%). Data visualizations were primarily designed for team use (12/18, 67%) rather than individual clinicians (4/18, 22%). Evaluation methods varied among asking the clinicians directly (11/18, 61%), observing user behavior through clinical indicators and use log data (14/18, 78%), and usability testing (4/18, 22%). The studies reported high scores on standard usability questionnaires, favorable surveys, and interview feedback. Improvements to underlying clinical indicators were observed in 78% (7/9) of the studies, whereas 22% (2/9) of the studies reported no significant changes in performance.

**Conclusions:**

This scoping review maps the current literature landscape on dashboards based on routinely collected clinical indicator data. Although there were common data visualization techniques and clinical indicators used across studies, there was diversity in the design of the dashboards and their evaluation. There was a lack of detail regarding the design processes documented for reproducibility. We identified a lack of interface features to support clinicians in making sense of and reflecting on their personal performance data.

## Introduction

### Background

Dashboards have evolved from single-view reporting information based on large raw data sets to customizable interfaces with multiple views and purposes, such as communication, learning, motivation, monitoring, and decision support [1]. The use of dashboards in many clinical settings has been well established [2]. Studies on these dashboards have focused on patient monitoring and clinical decision support using electronic medical records (EMRs), electronic audit and feedback (e-A&F) systems based on quality and safety standards, and management dashboards to support the day-to-day operations of departments. Evaluations of clinical dashboards tend to evaluate accuracy (decision-making), efficiency (time-to-task completion), usability (user satisfaction), and adherence to guidelines (quality and safety) [3-5].

There are known organizational, cultural, and technical issues with collecting and reporting on clinical indicators [6], sometimes referred to as quality or performance indicators. Despite the limitations of clinical indicator data, health professionals’ attitudes suggest that there is an appetite for easier and timely access to routinely collected clinical indicator data for personalized performance feedback [7]. Mainz et al [8] categorizes clinical indicators as *structural*, *process*, or *outcome indicators*. Structural indicators describe the type and number of resources by a health system or organization to deliver care, for example, the number of staff, patients, beds, and supplies. Process indicators measure the activities and tasks in patient care episodes, for example, patients were assessed by a physician within 24 hours of referral, and patients were treated according to clinical guidelines. Outcome indicators are states of health or events that follow care, which may be affected by health care. Mainz et al [8] proposes that outcome indicators are usually related to death, disease, discomfort, disability, and dissatisfaction. Clinical indicators can also be categorized as *generic* or *disease-specific*. Generic indicators measure aspects of care that are relevant to most patients (length of stay, readmissions, and late discharges). Disease-specific indicators are diagnosis-specific and measure specific aspects of diseases and conditions (hip fractures after the second operation and patients with lung cancer who are alive 30 days after surgery).

Reflective practice and lifelong learning are central to continuing professional development (CPD) frameworks mandated by medical boards around the world [9]. Participation in CPD programs ensures that medical specialists meet the standards required to maintain their specialist registration. Although board examinations and work-based assessments certify the initial competence of medical graduates, practicing clinicians require ongoing self-assessment to maintain standards and identify improvement needs [10]. Professional development frameworks often include references to the use of practice data for clinical audits and reflection [11,12].

Dashboards are commonly used for audit and feedback (A&F), an established process for improving professional practice by reviewing data based on existing benchmarks in the quality and safety literature [13]. Although there have been some successes, existing studies on e-A&F dashboards show that evidence is limited in terms of effectiveness for improving performance [14]. Furthermore, studies on dashboards designed to support clinician reflective practice and lifelong professional learning are scarce and heterogeneous [15].

This work aims to fill the gap in the literature on the use of data from disparate clinical sources to generate new insights that lead to practice reflection by clinicians.

e-A&F dashboards address known questions about clinical performance, whereas clinical *practice reflection* dashboards focus on presenting routinely collected data to clinicians to engage with and reflect on and to reveal new questions about their individual and wider team practice.

There has been some emerging literature on clinical practice reflection dashboards designed to support the reflective practice of clinicians [16]. This scoping review will summarize the literature on dashboards that support the reflective practice of clinicians and systematically map the features and outcomes of published interventions.

### Objectives

This scoping review aims to systematically map the different characteristics of feedback interfaces that support clinicians in reflecting on their practice. The data extracted from the included studies will provide insight into why the interfaces were created, how they were designed and evaluated, and what were the reported outcomes.

The scoping review was guided by the following 6 research questions (RQs):

- RQ1: What was the purpose of the performance feedback interfaces?
- RQ2: What clinical indicators were used and how are they visualized?
- RQ3: How were the interfaces designed?
- RQ4: What were the methods used to evaluate the interfaces?
- RQ5: How successful have the interfaces been?
- RQ6: What are the key design considerations for developing future interfaces?

## Methods

### Overview

The scoping review process was conducted following the methodology and guidelines by Arksey and O’Malley [17]. The process is outlined in 6 steps as follows: identifying the RQ; identifying the relevant studies; study selection; charting the data; collating, summarizing, and reporting results; and consultation.

To ensure the quality of the studies, the review only included studies published in peer-reviewed journals that had the full text available. Additional quality analysis was not conducted on the included studies, as quality assessment is not a requirement for a scoping review [18], and there are no established criteria to evaluate the quality of clinical dashboard studies. Quality assessment was not conducted to ensure that lessons were gained from a diverse range of work.

### Search Strategy

The search strategy was developed in consultation with the university librarian, using the *Population-Concept-Context* mnemonic [19].

The target population included any medical specialist as defined by the Australian Health Practitioner Regulation Agency registry of medical specialties and subspecialties, general practitioners (primary care physicians), and registrars (residents) in specialist medical training programs.

The review explored the concept of the use of clinical indicators to provide insight into a clinician’s own practice. Synonyms were generated for the search term clinical indicator, such as *quality indicator* and *performance indicator*. Generic terms for possible data sources for clinical indicators included search terms, such as *administrative, medical, and surgical data*. An additional concept focused on the intervention used in the study, that is, the feedback user interface. Synonyms for search terms included the following: *dashboard*, *visualization*, and *report*. Search terms, such as *feedback* and *reflection* were specifically not included to maximize the breadth of the search. We ensured that performance feedback and reflection dashboard studies were still captured in the search using the *clinical indicator* and *interface* search terms.

Peters et al [19] defines a context in terms of geographic location, setting, or cultural factors. No specific search terms were used for context, as there were no requirements related to the country of study, and hospital setting, such as public, private, inpatient, outpatient, rural, remote, or metropolitan.

The search strategy was developed by BB and was refined based on feedback from all the authors and the university librarian (JG). The search strategy was translated into a search query (Multimedia Appendix 1) and conducted on the following electronic databases: MEDLINE, Embase, Scopus (which includes IEEE Xplore and ScienceDirect), ACM Digital Library, and Web of Science. The electronic databases were selected to ensure coverage of clinical dashboard studies published in the fields of health informatics, data visualizations, and human-computer interaction research.

### Search and Study Selection

The initial search was conducted by BB. Articles were retrieved based on an agreed-upon search strategy. Next, BB screened all the retrieved article titles and abstracts against the inclusion and exclusion criteria. Concurrently, CP screened a random selection of 4.99% (184/3685) of the retrieved articles. BB and CP discussed the conflicts generated during the screening. The authors agreed to proceed to a full-text review after the first review returned 4.3% (8/184) conflicts from the abstracts screened.

Studies were eligible for this review if they met the following criteria: the study provided a medical practitioner with access to clinical indicator data to receive feedback on their performance, included details on the design and implementation of the interface, included information on the interface features (visual and functions), described the evaluation methods used, and was published in English in a peer-reviewed journal between 2010 and 2020.

Articles were excluded if the study participants were in medical school, as medical students were not considered as professional learners. Articles were also excluded if the intervention was designed for public health physicians and researchers, as the interface was concerned with data about communities and populations. If the full text was not available (eg, conference abstracts), the article was excluded. Articles that grouped a variety of medical practitioners were included. However, they were excluded if it was unclear which reported data and findings related to participants in our inclusion criteria.

### Data Extraction

Data were extracted from articles retrieved by BB and then reviewed by CP to mitigate bias. Table 1 maps the RQs to the descriptive data extracted from the included studies.

For RQ1, the purpose of each interface was extracted because there were no specialty or subspecialty restrictions on the search. By identifying the stated purpose and aims of each dashboard, we could better compare similar implementations. For example, dashboards for managing day-to-day operations are compared with dashboards for clinical quality improvement.

To understand the data presented in each interface, RQ2 extracted the names of clinical indicators (eg, length of stay), the data source (eg, EMR and clinical registry), and the technology used by the platforms. RQ2 also captured how the data were presented and the features of the interface by extracting design details, such as the data visualization types (eg, bar chart), interactivity (eg, zooming and filtering), and individual versus team views. We also identified the intended frequency of use of the interface. Kay et al [20] describes 2 mental systems that work differently and drive the way people think. System 1 performs *fast* intuitive thinking, which is automatic but can lead to bias and errors. In contrast, system 2 performs *slow* rational and logical thinking that is conscious and can override the initial insights acquired by system 1 [20]. We define *fast* use as at a glance or daily use. We define *slow* use as longer than a day (eg, weekly or monthly use).

RQ3 extracted the design process used in each study, as there is value in understanding how the interfaces were designed. We anticipate that the design approaches and methods used in these studies could be helpful for future researchers to design similar interfaces.

To address RQ4, we expected to see a diverse range of research methods conducted across controlled laboratory and authentic hospital settings. RQ5 then looked to assess which interfaces were effective in terms of usability, changes in practice, and patient outcomes. RQ4 and RQ5 together allow us to gauge the success of the studies in achieving their stated goals.

Finally, RQ6 identified the key factors to consider when designing interfaces to support the reflective practice of clinicians. RQ6 summarizes the practice points and recommendations proposed by the included studies.

**Table 1:** Research questions (RQs) and data planned to be extracted from included studies.

RQ		Data extracted
RQ1	What was the purpose of the performance feedback interfaces?	Stated purpose and aims
RQ2	What clinical indicators were used and how are they visualized?	Clinical indicators Visualization elements Frequency of intended use Individual or team use Static or interactive features Data source Technology
RQ3	How were the interfaces designed?	Design process
RQ4	What are the methods used to evaluate the interfaces?	Evaluation methods Laboratory vs in-the-wild settings
RQ5	How successful have the interfaces been?	Reported results and outcomes Strengths and limitations
RQ6	What are the key design considerations for developing future interfaces?	Practice points Recommendations

## Results

### General Characteristics of the Included Studies

The following section summarizes the general characteristics of the included studies, such as the year of publication, location of publication, citation trends, country of origin, specialty of participants, and study duration (Multimedia Appendix 2 [21-38]).

**Figure 1:**
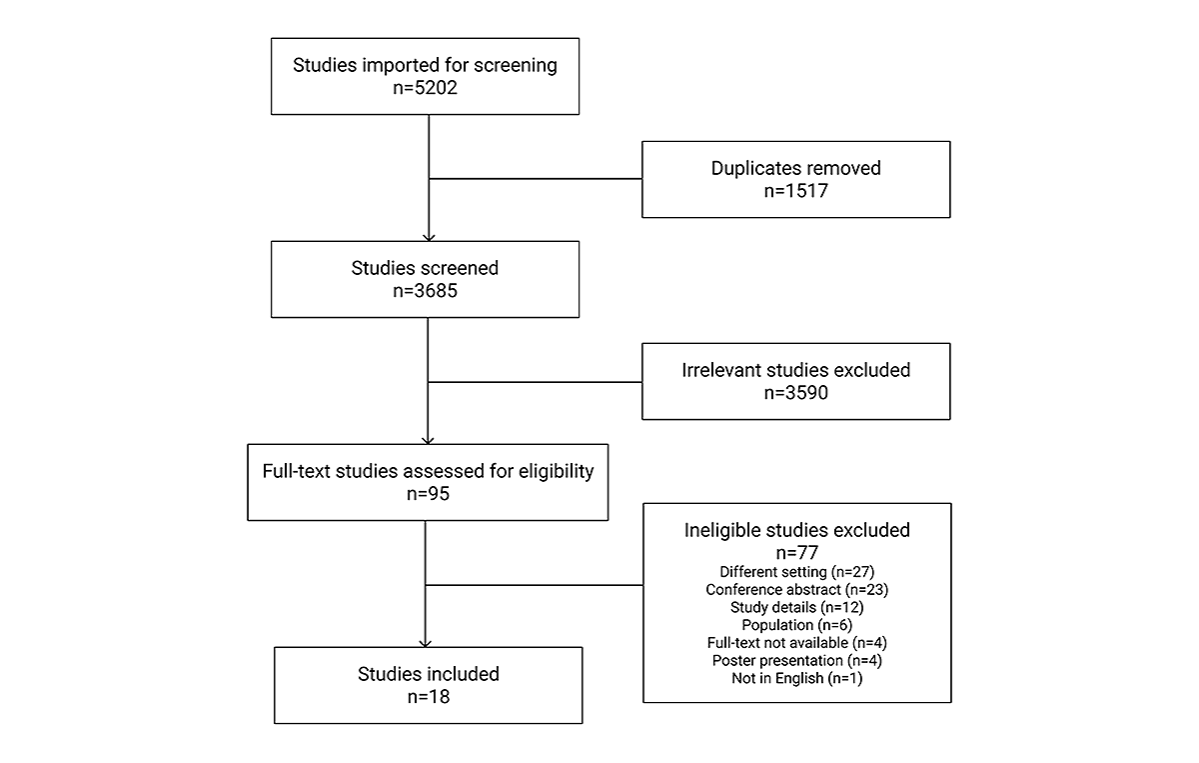
Flow diagram of search and selection studies.

Figure 1 shows the flow of articles from the identification, screening, and final inclusion. The original search conducted in November 2020 yielded 3685 potentially relevant citations after duplicates (n=1517) were removed. After title and abstract screening, 2.58% (95/3685) citations met the eligibility criteria, and the corresponding full-text articles were procured for full-text review. After reviewing all the full-text articles, 81% (77/95) studies were excluded according to the inclusion and exclusion criteria; 19% (18/95) dashboard studies remained and were included in the analysis.

Of the 77 excluded studies, 27 (35%) were excluded for having a different setting. Studies were identified as having a different setting if the interface was not used to provide feedback on individual or team performance, for example, used outside a hospital or clinic environment, such as public health researchers. In all, 8% (6/77) of the studies were excluded as the target participants of the study did not meet the inclusion criteria, for example, nurses, pharmacists, and medical researchers. Overall, 16% (12/77) of the studies were excluded owing to insufficient study details to be extracted for analysis. Studies were also omitted if the full study text was not accessible, for example, conference abstracts (23/77, 30%) and poster presentations (4/77, 5%). Of the 77 excluded studies, 4 (5%) could not be retrieved, and 1 (1%) study was excluded because it was not published in English.

All studies were published between 2010 and 2020, with 72% (13/18) published after 2015. Most of the citations (181/208, 87%) occurred after 2015. Linder et al [21] contributed 27.9% (58/208) of all the citations in the studies between 2010 and 2020.

Of the 18 selected studies, 3 (17%) studies [22-24] cited another study on an electronic health record (EHR) dashboard to improve antibiotic prescription [21]. Laurent et al [23] also cited a maternity dashboard pilot study [25]. Schall et al [24] cited an earlier study by the same authors on the evaluation of a health care information technology (HIT) dashboard based on quality indicators [26].

The countries of origin of these studies are summarized in Table 2. Most of the studies were conducted in English-speaking countries, with 55% (10/18) originating from the United States.

**Table 2:** Country of origin from included studies (N=18).

Country of origin	Count, n (%)	References
United States	10 (56)	[21,22,24,26-32]
Australia	2 (11)	[33,34]
Other (Canada, France, the Netherlands, Oman, Sweden, and United Kingdom)	6 (33)	[23,25,35-38]

By looking at the study participants, we could identify the specialty and subspecialty groups that use interfaces to engage with data about their performance. The participants of the included studies came from 11 distinct medical specialties or subspecialties, with 22% (4/18) of dashboard studies focusing on primary care physicians (or general practitioners). Studies have also evaluated dashboards for anesthesia (3/18, 17%). Of the 18 included studies, 3 (17%) studies did not specify a particular specialty or subspecialty of the participants, and 1 (6%) study included registrars (residents) who were still in specialist training programs.

In all, 50% (9/18) of the studies did not specify the duration of the evaluation. Of these studies, 56% (5/9) of the studies were deployed and evaluated in real-world hospital environments, and 44% (4/9) of the studies were conducted in controlled laboratory settings. The evaluation study duration ranged between 2 months [23] and 42 months [27].

### RQ1: Purpose

As the studies were conducted across a range of medical specialties, the purpose for each dashboard was also diverse.

Table 3 shows that the clinical dashboards in the review fell evenly across 3 categories. Performance improvement dashboards aim to present data to an individual or team to reflect on their practice and identify areas to change. Quality and safety dashboards track the agreed-upon clinical guidelines and benchmarks. They can be modeled with existing clinical practice improvement models, such as Plan-Do-Study-Act [39]. Management and operations dashboards are targeted to administrators and directors of departments to support the day-to-day functions of health care services. Laurent et al [23] was categorized as supporting quality and safety as well as management and operations.

**Table 3:** Purpose of included dashboard studies grouped by category (N=18).^a^

Purpose	Count, n (%)	References
Performance improvement	9 (50)	[27-31,33,36-38]
Quality and safety	6 (33)	[21,23-25,32,35]
Management and operations	4 (22)	[22,23,26,34]

^a^Included studies may be in more than 1 category.

### RQ2: Common Features

The following section summarizes the common clinical indicators used across the dashboards, how the indicators were presented to the end users, where the indicators were sourced, and the technology platform details. By identifying the lower-level data used and the functionality of each dashboard, we can see how the researchers aimed to fulfill the purpose of their feedback interface.

#### Clinical Indicators

As shown in Table 4, the use of clinical indicators varied across the studies. The study by Clark et al [34] was the only study that evaluated a dashboard that presented structural indicators to clinicians, such as consultant workload and bed availability. Process indicators were used in all but one of the studies. The most used generic indicators across the studies included the following: length of stay (7/18, 39%), readmission (4/18, 22%), and discharge (3/18, 17%), whereas *% acute respiratory infections (ARI) visits with antibiotics*, *Lymphedema index (L-Dex)*, and *Number of Atrial fibrillation (AF) diagnosis over time* were examples of specialty-specific indicators.

Of the 18 studies, 3 (17%) studies presented outcome indicators—mortality [23], patient complaints [25], and patient satisfaction [30].

**Table 4:** Clinical indicators by type from included studies (N=18).^a^

Clinical indicators		Count, n (%)	References
**Classification**			
	Structural	1 (6)	[34]
	Process	17 (94)	[21-38]
	Outcome	5 (28)	[23,26,30,36,38]
**Specificity**			
	Generic	15 (83)	[22-32,34-37]
	Disease-specific	3 (17)	[21,33,38]

^a^Included studies may have more than 1 type of clinical indicator.

#### Dashboard Presentation

The types of visualization used to present the underlying clinical indicators are summarized in Table 5. A combination of bar charts, tables, and line charts were used in 50% (9/18) of the studies. Gude et al [36] and Weiner et al [22] used all the 3 techniques.

Table 6 shows that most (12/18, 67%) of the dashboards evaluated were intended for team use, whereas 22% (4/18) of the dashboards were for individual use. Of the 18 dashboards evaluated, 2 (11%) dashboards were designed for both team and individual use. Clinicians work in specialty care teams, multidisciplinary teams, and as individual consultants; therefore, dashboard interfaces should show the relevant data depending on the setting.

The intended frequency of use of the dashboards was evenly split between fast and slow use as shown in Table 7. One urology dashboard was designed specifically for rapid or at-a-glance use [37]. In all, 17% (3/18) of the dashboards were designed for slow use and were reviewed every month [25,27,28]. Overall, 22% (4/18) of the included studies designed dashboards for slow use but did not specify the exact cadence for reviewing data [21,33,36,38].

**Table 5:** Dashboard visualization elements used in included studies (N=18).^a^

Visualization elements	Count, n (%)	References
Bar chart including histogram	10 (56)	[21-23,27,29,32-34,36,37]
Table	9 (50)	[22-27,31,36,38]
Line chart	9 (50)	[22,29,31,32,34-38]
Scatter plot	1 (6)	[35]
Meter	1 (6)	[22]
Radar including radial or spider-web	1 (6)	[30]
Pie chart including donuts or rings	1 (6)	[29]

^a^Included studies may have more than 1 visualization element.

**Table 6:** Dashboard designed for team or individual use (N=18).

Use	Count, n (%)	References
Team	12 (67)	[22-29,32,34,37,38]
Individual	4 (22)	[21,30,35,36]
Both	2 (11)	[31,33]

**Table 7:** Dashboard studies designed for fast or slow use (N=18).

Use		Count, n (%)	References
**Fast**			
	Daily	8 (44)	[22-24,26,29,31,32,34]
	Rapid or at-a-glance	1 (6)	[37]
**Slow**			
	Weekly	1 (6)	[30]
	Monthly	3 (17)	[25,27,28]
	Quarterly	1 (6)	[35]
	No details	4 (22)	[21,33,36,38]

#### Data Sources

Of the 18 included studies, 5 (28%) studies were conducted on top of the EMR and EHR systems, 4 (22%) studies were integrated with an existing data warehouse within the hospital infrastructure, 2 (11%) studies were integrated with the patient administration systems within the hospital, and 1 (6%) study used data from a clinical registry. In all, 44% (8/18) of the studies did not specify the data source used to implement the dashboard solution.

#### Technology

Overall, 22% (4/18) of the dashboard studies leveraged web development technologies, such as HTML, Cascading Style Sheet, and JavaScript. Open-source libraries, such as jQuery (OpenJS Foundation), D3.js (Mike Bostock), and HighCharts (Highsoft AS) were also used. Of the 18 studies, 4 (22%) studies used out-of-the-shelf enterprise solutions (SAS, Tableau, and Qlikview). In total, 11% (2/18) of the dashboard studies presented data using Microsoft Excel. In all, 33% (6/18) of the included studies did not specify the technology tools and platforms used to implement the dashboard solution.

Dashboards can be interactive, allowing users to engage with the data in multiple ways rather than a single static view. Interactive dashboards enable users to drilldown to gain background information, show comparisons, and highlight anomalies in the data visualizations [40]. Shneiderman et al [41] has described common features of advanced graphical user interfaces, including an overview of the entire collection of data, zooming into interesting items, filtering out uninteresting items, and retrieving additional details on demand.

Table 8 shows that most (15/18, 83%) of the dashboards were interactive, 11% (2/18) of the dashboards were static, and 6% (1/18) of the studies did not provide details on whether the dashboard was interactive or static [28].

**Table 8:** Dashboard studies designed to be interactive or static (N=18).

Interface design	Count	References
Interactive	15 (83)	[21-24,26,27,29,31-38]
Static	2 (11)	[25,30]
No details	1 (6)	[28]

### RQ3: Design Process

Only 56% (10/18) of the studies provided details on the design process used. User-centered design (3/18, 17%), co-design (2/18, 11%), and iterative processes (2/18, 11%) were the specific approaches mentioned in the papers. Mulhall et al [35] used both co-design and user-centered design approaches. An iterative and user-centered design was used by Stattin et al [37]. Although the remaining 44% (8/18) of the studies had no details of the design processes, 55% (10/18) of the studies did report details used a diverse range of methods, including focus groups, interviews, workshops, and process mapping.

### RQ4: Evaluation Methods

The evaluation methods, grouped by type, are listed in Table 9. A mix of quantitative and qualitative research methods were used. Majority of the studies (10/18, 57%) quantitatively evaluated the impact of the dashboards. Data were primarily sourced from EMRs, clinical registries, and patient administrative systems. Questionnaires, such as pre- and postsurveys, standardized single ease questions (SEQ) and system usability scale (SUS) were used in 44% (8/18) of the studies. Methods used in the remaining studies included analysis of access logs (3/18, 17%), formal cluster randomized control trials (2/18, 11%), case studies (2/18, 11%), interviews, the think-aloud protocol, heuristic evaluation, and eye tracking.

In terms of the evaluation setting, most of the studies (13/18, 72%) were conducted in authentic settings, such as in the emergency department, primary care clinics, or hospital inpatient wards, whereas the remaining studies (4/18, 22%) were conducted in controlled laboratory settings.

**Table 9:** Evaluation methods used by included studies (N=18).^a^

Method		Count, n (%)	References
**Asking the users**			
	Questionnaires or surveys	9 (50)	[23,24,26,28,31,33,35,36,38]
	Interviews	2 (11)	[33,38]
**Evaluating user behavior**			
	eHealth data analysis	10 (56)	[21,22,27-31,34,36,37]
	System usage log analysis	4 (22)	[21,27,29,35]
**Evaluating usefulness of the interface**			
	Expert method	1 (6)	[26]
	Usability user study	3 (17)	[24,33,38]

^a^Included studies may have more than 1 evaluation method.

### RQ5: Reported Outcomes

The reported outcomes of each of the included dashboard studies are summarized further (Multimedia Appendix 3 [21-38]). The methods were grouped by (1) direct feedback from end users, (2) data analysis of the underlying eHealth data, (3) data analysis of platform use logs, (4) expert usability evaluation, and (5) usability testing with end users.

#### Asking the Users: Questionnaire, Survey, and Interview

Studies that asked for feedback on the dashboard directly from end users using standard questionnaires, surveys, and interviews are summarized in Table 10.

Of the 18 included studies, 5 (28%) studies used a standardized questionnaire to gauge the individuals’ assessment of dashboard usability. SUS is a validated questionnaire that measures users’ overall satisfaction with a graphical user interface [42]. The questionnaire is interface agnostic and consists of 10 items with total scores ranging from 0 to 100 [43]. In all, 17% (3/18) of the included studies reported high mean SUS scores of 82.6 (SD 11.5) [23], 83 (SD 7.6) [26], and 87.5 (SD 9.6) [24]. Overall, 6% (1/18) of the studies reported a median SUS score of 73.0 (SD 15.0) [38].

In addition to the SUS questionnaire, Schall et al [24] also conducted a Post-Study System Usability Questionnaire (PSSUQ). The PSSUQ consists of 19 items that measure users’ perceived satisfaction with a product [44]. The questionnaire consists of three subscales as follows: system usefulness, information quality, and interface quality. The study found an overall mean PSSUQ score of 1.7 (SD 0.5) with subscale scores of 1.5 (SD 0.4), 1.8 (SD 0.8), and 1.8 (SD 0.8)—suggesting the dashboard had good usability.

**Table 10:** Reported results from standardized questionnaires, surveys, and interviews (N=18).

Evaluation method	Reported outcomes	References
Standardized questionnaire	Mean SUS^a^ score of at least 73.0 across 5 studies (range 73.0-87.5). PSSUQ^b^ score of 1.7 (SD 0.5). All tasks rated median SEQ^c^ score of 1 (very easy) or 2 (easy).	[23,24,26,33,38]
Survey	Respondents had favorable responses to the dashboards (range 72-79). Respondents stated the data were actionable (range 48-69). Respondents felt the data improve their practice (range 64-98).	[28,29,31,35]
Interview	Interviewees were interested and enthusiastic about the individual patient dashboard. Interviewees were generally excited to have the opportunity to see the cohort dashboard but commented on its complexity. Interviewees were generally positive about the clinical performance summary, patient lists, suggested actions, and detailed patient-level information views. Interviewees identified improvements on the clinical performance summaries view (eg, inclusion of CIs with differing guidance was confusing).	[33,38]

^a^SUS: system usability scale.

^b^PSSUQ: Post-Study System Usability Questionnaire.

^c^SEQ: single ease question.

Janssen et al [33] asked 5 participants to rate the ease of completing tasks on the lymphedema dashboards using a 7-point SEQ [45]. All tasks on the individual dashboard received a median SEQ rating of either 1 (very easy) or 2 (easy). Similarly, all tasks on the cohort dashboard received a median SEQ rating of 1 or 2. The last task on the cohort dashboard was attempted by only 3 participants and received a median SEQ rating of 3.

User feedback surveys were conducted in 17% (3/18) of the studies. Mulhall et al [35] surveyed 316 family physicians who used the quality improvement dashboard in long-term care practice. The overall quality of the dashboard was rated as *good* (45%) and *very good* (34%), and 69% of physicians said they were *likely* or *very likely* to implement one of the suggested changes.

Khanna et al [28] surveyed 48 primary and specialty care practices on their perceptions of a practice transformation analytics dashboard as a tool to present data that are actionable in health care design. The study found that 96% of surveyed practices reported having previously reviewed their cost data, 72% had favorable responses to the dashboard, and 48% found dashboard data actionable (n=25).

Ehrenfeld et al [31] evaluated the perceptions of anesthesia residents on a performance feedback dashboard. The study found that 91% of respondents said they would like to receive a systematic review of practice performance data every 1 to 4 weeks (n=48), whereas 98% of resident respondents said they could improve in at least one and often multiple areas. Only 10% of the respondents believed that they were compliant in all 6 areas listed. All respondents, except 1, noted that they would like to receive feedback in some electronic form, for example, emails, websites, and smartphones.

#### Evaluating User Behavior Through eHealth Data Analysis

Table 11 summarizes the key results related to changes in the underlying clinical indicators and the dashboard use logs.

**Table 11:** Reported outcomes from data analysis of eHealth data and system use logs.

Evaluation method	Reported outcomes	References
eHealth data analysis	2 out of 9 studies evaluating eHealth data reported positive changes to CI data. 2 out of 9 studies reported no change to CI data.	[21,22,27,29,30,34-37]
System use log data analysis	>50% of participants viewed the dashboard in 2 studies (range 28-50). A median of 55 views from 30 users was observed in 1 study.	[21,29,35]

Hester et al [27] observed improvements in emergency department balancing measures, which included a higher emergency department discharge rate (70.7% vs 72.8%; *P*=.05), lower charges (ratio 1:0.86; *P<*.001), shorter length of stay (2.9 hours vs 2.6 hours; *P*=.001), and lower 7-day revisit rates (15.4% vs 11.6%; *P<*.001). Inpatient charges decreased (ratio 1:1.14; *P*=.01), but the length of stay and readmission remained stable.

Patel et al [29] observed that the composite discharge mix index improved during the 5-month study period; they observed a 79.3% completion rate in the intervention group (n=537) compared with 63.2% in the control group (n=516).

In 53.8% of the cases (n=288), Gude et al [36] observed that intensive care specialists overestimated their clinical performance, whereas in 13.5% of the cases, they underestimated their performance. Participants overestimated peer performance and set targets 20.3% higher than the top performance benchmark. In 68.4% of the cases, intentions to improve practice were consistent with actual gaps in performance (without feedback); it increased to 79.9% after receiving feedback. In 56.3% of the cases, participants still wanted to improve the aspects that they were already top performers in, and in 8.3% of the cases, they lacked improvement intentions, as they did not consider indicators important.

Stattin et al [37] evaluated a SMART (specific, measurable, accepted, realistic, timely) performance feedback dashboard based on data from a national cancer registry. The proportion of patients reported in a timely fashion to the registry increased from 26% in 2011 to 40% in 2013 (*P<*.001). The use of active surveillance for men with very low-risk prostate cancer increased from 63% to 86% (*P<*.001). The waiting time remained long. In 2013, the overall median time from receipt of referral to the first visit to a specialist clinic was 35 days (IQR 21-58). From prostate biopsy to the date when the patient received information on their cancer diagnosis was 29 days (IQR 21-40).

Weiner et al [22] evaluated a dashboard for leadership to monitor emergency physicians’ and radiologist’s performance against established targets. They found that acute patients’ (who may require admission) monthly length of stay dropped by 54 minutes. Similarly, the monthly length of stay of lower acuity patients (outpatients) dropped by nearly an hour. Finally, the number of patients in the emergency department who left without being seen fell from 165 per month to 10 per month.

Clark et al [34] observed improvements in process indicators during a 3-month intervention of a clinical dashboard that supported decision-making. The indicator performance improved by an average of 21.2% across the 5 indicators (range 8-38). In particular, discharge plans communicated to patients 24 hours before discharge increased from 48% to 86%. In addition, pharmacy scripts written 24 hours before patient discharge increased from 62% to 84%.

Linder et al [21] investigated whether an acute respiratory infection dashboard changed prescription rates. The study found no difference between intervention and control practices in antibiotic prescriptions for all acute respiratory infection visits.

#### Evaluating User Behavior Through System Access Log Analysis

Mulhall et al [35] evaluated a dashboard to support primary care physicians in quality improvement. The study found that 50% of the general practitioners viewed the web-based report (n=400), with 90% signing up for email delivery. Participants who viewed at least one of their reports had an almost 2% reduction in antipsychotic prescribing rates.

Patel et al [29] conducted a cluster randomized controlled trial to evaluate a dashboard to support team-based A&F. During the 5-month intervention period, the dashboard was accessed 104 times by 40 users in February, 77 times by 33 users in March, and 55 times by 30 users in April. During the washout period, the dashboard was accessed 48 times by 20 users in May and 48 times by 24 users in June. After a 9-month intervention period, the use logs showed that 28% of clinicians used the dashboard at least once (n=72); these clinicians had lower overall acute respiratory infection prescribing rates (42%) compared with the control group (50%; *P*=.02) [21].

#### Expert Usability Method

Only 6% (1/18) of the studies conducted a heuristic evaluation of the dashboard interface [26]. In all, 2 human factor professionals and 3 focus group members evaluated the dashboard based on Nielsen 10 heuristics [46]. The expert review identified 20 suggestions for the changes. Overall, 5 changes were recommended by 40% or more of the evaluators. The top suggestion (with 60% of evaluators in agreement) was to include a cover sheet documenting the goal of the program and quality indicator criteria (clarity) and to remove the catheter quality indicator (repetitive).

#### User Study Usability Methods

The key results from the studies that involved end-user usability testing of the interfaces were generally positive. These studies required participants to complete predefined tasks on the interface to identify errors and measure the time to completion.

Janssen et al [33] found that 5 participants completed all the think-aloud protocol tasks on the individual patient dashboard (n=5). On the cohort dashboard, only 1 of the 5 participants was able to complete the first task to identify the proportion of patients with lymphedema that had >10 resected nodes. The last task on the cohort dashboard, which required participants to identify the proportion of patients within the organization having ongoing treatment for lymphedema and a BMI in the overweight range, was only attempted by 3 participants.

Brown et al [38] evaluated an e-A&F dashboard to understand the optimal interface design for the clinical A&F process. In all, 7 participants identified a median of 10 errors (range 8-21). A median of 5 tasks were completed out of the 7 evaluation tasks (range 4-7); 16% (6/38) of the possible heuristic categories were violated, with the most frequently violated being *workflow integration* (n=40).

Schall et al [24] observed that the time on task improved in 6 of the 8 evaluation tasks between the conventional and HIT dashboards (n=6). In terms of accuracy, the tasks completed without errors improved across 5 of the 8 tasks. Task completion without errors remained the same between the conventional and HIT dashboards in the first 2 evaluation tasks. Tasks completed without errors decreased in 1 evaluation task (pressure ulcers).

### RQ6: Future Design Considerations

There were 4 key themes that were identified across the included studies related to future dashboard design considerations.

#### Engagement With Clinical Staff

A key design consideration was the involvement of end users throughout the development process. For example, Laurent et al [23] followed a user-centered process when developing a tool to guarantee usability and ensured that the information displayed did not lead to misunderstandings or interpretation errors.

Promoting dashboards through demonstrations at meetings with individuals or teams was suggested by Schall et al [24] as a strategy to engage clinical staff. To fully integrate the dashboard use in practice, the study suggested updating practice reminders, providing actionable feedback of quality improvement data, and reporting to senior leaders. In addition, local champions or change agents in each unit were responsible for using the dashboard during interprofessional daily huddles.

#### Clinical Indicators

The selection of clinical indicators was a common topic discussed in the included studies. Stattin et al [37] noted that the selection of quality indicators should be based on recently published guidelines that have been widely accepted. Patel et al [29] highlighted that to be effective in improving care, the use of process indicators that the evidence trying to measure an outcome is continuously evaluated, and providers have the opportunity to provide feedback on how meaningful they find the measures. The principle of fairness should also be considered when selecting clinical indicators; specifically, performance standards need to be evaluated and set concerning quality care, for example, the minimum standards for competency in residency programs [31].

When presenting clinical indicators to clinicians, Brown et al [38] suggested that indicators should be framed positively where appropriate to emphasize achievement. In addition, clinical indicators should be prioritized automatically.

Linder et al [21] highlighted that reporting clinical indicators, by itself, is frequently insufficient to improve the quality of care. Linder et al [21] suggested that quality reporting likely needs to be coupled with other interventions, such as *clinical detailing, clinical decision support, patient education, or financial incentives*. Clark et al [34] also suggested cointerventions, such as a dashboard, including decision-support tools.

Herzke et al [30] highlighted the benefits of attributing performance data to individual clinicians rather than admitting clinicians. However, the authors warned that the computational requirements of their methodology were not trivial and required linking billing data with administrative patient-level data, which may be challenging to operationalize.

Gude et al [36] proposed that more intensive measures, such as verbal feedback and feedback discussions in teams rather than among individuals might be required to ensure clinicians recognize the importance of indicators and trust in data.

#### Support to Interpret Performance Data

The studies identified clinicians having difficulty interpreting the clinical indicator data to make sense of their individual and team performance. To support clinicians in interpreting their data, Schall et al [26] suggested that if quality indicator scores do not have meaning, the score should not be included. If included, more precise definitions of symbol color and quality indicators would be helpful. Similarly, a cover sheet documenting the goal of the program and quality indicator criteria was also proposed.

To prevent benchmarks from being perceived as unrealistically high, Gude et al [36] recommended delivering multiple performance comparators, such as median, top 10% peer performance, and own past performance. Ranking individual provider performance relative to peers was also suggested by Herzke et al [30].

Brown et al [38] proposed comparing scores of users to desirable performance labels, such as using a traffic light system to reduce ambiguity. If the dashboard presents suggested actions, it should provide further data analysis and visualization related to recommended improvement actions and clearly explain what performance data specifically refer to.

To address the known issues around attributing performance data between admitting and consulting clinicians during the episode of care, Herzke et al [30] found that ensuring that data can be credibly attributed to the individual provider was integral in dashboard design.

Dashboards should have the ability to provide details on demand related to why particular improvement actions were suggested, how they have been implemented in other organizations, and patient-facing information [38]. For intermittent dashboard use, Janssen et al [33] suggested it may be helpful to add scaffolding to support exploration of key aspects of practice performance and a history mechanism to enable clinicians and administrators to track progress and changes.

#### Technology

Broader technology considerations were also highlighted in the included studies. Stattin et al [37] described a scenario in which clinicians may not log in to the dashboard platform. Emails should distribute quarterly reports to department heads to support clinicians’ adoption of new technology.

Looking into the future of dashboards based on repurposed clinical indicator data, Clark et al [34] outlined the need for dashboards to continue to focus on quality metrics and to include decision-support tools. In addition, Clark et al [34] predict that initiatives that focus on improving patient experience, such as patient-reported satisfaction, will feature on future dashboards incorporating predictive modeling within dashboards to provide a broader set of information for clinicians.

#### Continuing Professional Development

Activities completed by clinicians involving reviewing their performance and measuring patient health outcomes are considered CPD activities in specialist professional performance frameworks.

For dashboards that include suggested improvement actions, Brown et [38] suggested allowing clinicians to add their own actions, which should be saved automatically. Clinicians should also be allowed to easily save, mark actions as implemented, and view those of other users within their organization.

Mulhall et al [35] identified the added benefits of an e-A&F dashboard. These reports can be used as part of a self-reflective study toward continuing medical education credits required in Ontario [35].

## Discussion

### Principal Findings

#### Overview

The results of this scoping review summarized and mapped the existing literature on emerging performance feedback dashboards based on routinely collected clinical indicator data. The scoping review adds to the literature in several ways. First, the review provides an overview of the different contexts in which these interfaces are used. Second, the review identified common visual and functional features. Third, this review summarizes the design processes and evaluation methods. Finally, the review reports the key outcomes of the included studies and the future design considerations proposed by the authors.

The following section discusses the review implications with respect to the initial RQs.

#### RQ1: Purpose

##### The Purpose of the Dashboards Included Performance Improvement, Quality and Safety, and Management of Operations

Performance or quality improvement interfaces are focused on presenting relevant clinical indicator data to allow clinicians to reflect on their individual and team performance.

##### There Is Potential to Improve Support for Interpretation

Dashboards may have scaffolding questions to support a clinician’s metacognitive processes and suggest improvement actions to implement. However, only 2 studies [35,38] have used these techniques to support the end users interpret their performance data. Guidance for clinicians to make sense of their performance data was a common theme identified across the included studies.

#### RQ2 and RQ3: Common Features and Design Processes

##### Generic Indicators Dominated the Studies

Most of the underlying clinical indicators used to populate the dashboards were categorized according to Mainz et al [8] as *process* and *generic* indicators. Length of stay, 28-day readmissions, and late discharges measure the activities in episodes of care. Generic indicators are not only relevant to specific specialties or subspecialties. Generic process indicators seem to be suitable indicators, as most of the dashboards were designed for team use in a specialty craft group or a multidisciplinary team.

##### Most Dashboards Were Designed for Group Use

As Herzke et al [30] reported, it is difficult to attribute the performance of individual clinicians when multiple consultants can interact with a patient during a single episode of care. Therefore, it is important for the whole team responsible for outcomes to see the performance indicators and work together to review that information.

##### The Studies Had a Similar Number of Fast and Slow Dashboards

The dashboards were evenly split for fast (<1 day) and slow use (weekly, monthly, or quarterly). Dashboards designed for emergency, intensive care, and maternity wards tend to be for fast use, where data should be optimized for constant monitoring and legible at a glance. Slow dashboards emphasized changes in clinical indicators over time, comparisons with peers, and reflection and goal-setting features.

##### Clinicians Were Engaged During the Design Process, but Details Were Often Unclear for Reproducibility

Although some of the studies in this review used user-centered and co-design approaches, most studies did not provide details on how their interfaces were designed. Without a description of the design approach and methods used, it is difficult for the studies to be replicated in future studies. By conducting user research and involving clinicians in the design process, HIT projects shift the role of designers from being experts to facilitators of the design process [47]. End users, such as clinicians, medical administrators, nurses, and allied health practitioners are empowered to engage in the design process. Ultimately, researchers gain a deeper understanding of the context of end users and create solutions that address real-world problems. Increased staff engagement was evident in the study by Mulhall et al [35], where the authors used co-design methods to develop a dashboard to improve prescribing rates. They observed a 2% reduction in antipsychotic prescriptions, and most of the participants (n=316) stated that they liked the dashboard and were likely to implement suggested practice changes.

#### RQ4 and RQ5: Evaluation Methods and Reported Outcomes

##### Dashboards Were Evaluated Either in a Controlled Laboratory Setting or an Authentic in-the-Wild Environment

Laboratory studies, such as usability testing, allow researchers to identify whether users are able to complete intended tasks on the interface with minimal errors. The use of standardized usability questionnaires allows researchers to compare utility and satisfaction scores among similar studies. On the other hand, in-the-wild studies allow researchers to identify adoption and implementation issues as the intervention is deployed in authentic environments, such as emergency wards and primary care clinics. Researchers are able to identify changes in actual performance by analyzing eHealth data in EHRs and patient administration systems. Analyzing system access log data provides another perspective on user behavior, allowing researchers to compare how participants thought they used the system with their actual use patterns.

##### Overwhelmingly Positive Responses From Participants on Dashboard Usefulness and Ease of Use

The standardized questionnaires, surveys, and interviews revealed that most participants found the dashboards to be useful and easy to use. Although the 5 studies reported high usability scores [23,24,26,33,38], none of the studies investigated changes in the underlying clinical indicators before and after clinicians used the dashboard in practice. Future dashboard studies should consider conducting a mix of controlled laboratory user testing and in-the-wild studies to understand the users’ initial reaction to an interface and its impact on practice.

##### A Majority Showed Promising Results, Even for the Small Set of in-the-Wild Studies That Assessed Improvements to Clinical Indicators

Only 22% (2/9) of studies reported no significant change in clinical indicators after the intervention period, whereas 78% (7/9) reported improvements. In all, 22% (2/9) of studies [21,29] revealed that <50% of the participants in their study accessed the dashboard during the evaluation period, suggesting issues with implementation and adoption. Although the studies evaluated the dashboards in either a controlled laboratory or authentic in-the-wild environment, no study evaluated the dashboard in both settings. Studies evaluating the impact of dashboards on clinical performance can be improved by incorporating data collected from controlled and authentic settings. Laboratory studies provide insight into the usability (accuracy, efficiency, and satisfaction) of interfaces for target users to achieve specific goals. Authentic in-the-wild studies provide insight into whether the interface supports changes in individual practice, internal processes, and patient health outcomes. Together, these environments provide rich data on the usefulness and effectiveness of the interface.

#### RQ6: Future Design Considerations

##### Engaging Clinicians During the Design Process Is Integral in Successful Implementation of Dashboards

The key themes identified related to future dashboards focus on the importance of engaging with staff in the design process, selecting and presenting appropriate clinical indicators, and supporting clinicians in interpreting performance data. Clinical staff must be involved in the design process of dashboards to support reflection on practice. The design should consider the differences in how care teams work, such as individual and team displays, frequency of use (at-a-glance vs long-term use), and specialty specific clinical indicators. The indicators selected must be relevant to the clinician’s work and presented to maximize understanding. Aligning the design of the dashboard interfaces to existing clinical performance improvement frameworks, such as Plan-Do-Study-Act [39], could better support clinicians in interpreting their individual and team performance data. Some techniques that could support this process of sense-making and reflection include scaffolding questions and annotation features with private notes [33].

##### Scaffolding Reflection to Support Long-term Professional Learning

The articles largely evaluated the usability and usefulness of the performance feedback interfaces. The current set of articles in this review suggests that the dashboards were relevant to their practice and that clinicians had a strong understanding of the clinical indicator data presented, both important steps in the reflection process. The interfaces received positive results related to the self-monitoring of performance data. However, the interfaces lacked features designed to support metacognitive processes, such as self-reflection, planning, and goal-setting [48]. One study incorporated features to suggest improvement actions based on guidelines and the ability to save personal improvement actions [38]. There is an opportunity to better understand how clinicians and teams make sense of their performance data, particularly how clinicians conclude that current practice is appropriate, when to initiate change in behavior, or conclude that past practice change initiatives have been effective. Clinicians may undertake improvement actions, such as conducting an A&F project, peer mentoring, or upskilling through the completion of CPD activities.

### Limitations

The review was restricted to specific databases (MEDLINE, Embase, Scopus, ACM Digital Library, and Web of Science), and a defined search query. This search strategy breadth was refined in consultation with the authors and the university librarian to ensure that the search captured articles across the health informatics, human-computer interaction, and data visualization fields.

As a result, the review does not include articles indexed by CINAHL, Cochrane, PsycINFO, and ERIC. The review does not include gray literature because no quality assessment of studies was planned to be conducted; therefore, the search was restricted to peer-reviewed articles only. Despite care in the design of the search process, some studies may not have been captured owing to their journal or indexing bias.

The search was restricted to a specific time frame to ensure the review was feasible to conduct, which could have led to some older studies being excluded. Limiting the time frame to the last 10 years ensured that the review captured changes in technology. The time frame also focused on state-of-the-art case studies rather than on the history of clinical dashboards.

The review followed the guidelines by Arksey and O’Malley, where a quality assessment of the studies is not required. As such, the review identifies a breadth of research, although this includes work that may not have been validated.

### Conclusions

Our work was motivated by the need for effective tools that support clinicians in reflecting on their practice. This scoping review mapped the current landscape of literature on dashboards based on routinely collected clinical indicator data to support reflection. Although there were common data visualization techniques and clinical indicators used across studies, there was variance in the design and evaluation of the dashboards. We identified a lack of interface features to support clinicians in making sense and reflecting on their personal performance data. We conclude that there is a gap in the literature on dashboards based on routinely collected clinical indicator data that are personalized and scaffolded visualization interfaces to support long-term reflection.

## Appendix

Multimedia Appendix 1 Example MEDLINE search terms.

Multimedia Appendix 2 Summary of characteristics of studies included in the scoping review.

Multimedia Appendix 3 Summary of results of studies included in the scoping review.

## Abbreviations

A&F: audit and feedback
CPD: continuing professional development
DHCRC: Digital Health Cooperative Research Centre
e-A&F: electronic audit and feedback
EHR: electronic health record
EMR: electronic medical record
HIT: health care information technology
PSSUQ: Post-Study System Usability Questionnaire
RQ: research question
SEQ: single ease question
SMART: specific, measurable, accepted, realistic, timely
SUS: system usability scale
