# Supplementary material for: State-of-the-art Dashboards on Clinical Indicator Data to Support Reflection on Practice: Scoping Review
Source: JMIR Med Inform. 2022 Feb 14;10(2):e32695. doi: 10.2196/32695 (PMC8887640; doi:10.2196/32695)
Supplement: Multimedia Appendix 1 [file medinform_v10i2e32695_app1.docx]

## Multimedia Appendix 1

MEDLINE (Ovid, 2010 to Nov 2020)

| # | Searches |
| --- | --- |
| 1 | ("medical practitioner*" or "health practitioner*" or "health professional*" or "medical specialist*" or clinician* or doctor*).mp. |
| 2 | exp Physicians/ |
| 3 | physician*.mp. |
| 4 | exp Pediatrics/ |
| 5 | p?ediatrician*.mp. |
| 6 | anesthetists/ or anesthesiologists/ |
| 7 | (an?esthetist* or anesthesiologist*).mp. |
| 8 | ("addiction medicine specialist*" or neonatologist* or haematologist* or immunologist* or microbiologist* or cardiologist* or "clinical geneticist*" or "clinical pharmacologist*" or endocrinologist* or gastroenterologist* or hepatologist* or geriatrician* or immunologist* or allergist* or oncologist* or nephrologist* or neurologist* or rheumatologist*).mp. |
| 9 | (dermatologist* or "general practitioner*" or intensivist* or "hospital administrator*" or "medical administrator*" or obstetrician* or gynaecologist* or "gynaecological oncologist*" or urogynaecologist* or ophthalmologist* or pathologist* or psychiatrist* or radiologist* or surgeon* or neurosurgeon* or otolaryngologist* or urologist*).mp. |
| 10 | 1 OR 2 OR 3 OR 4 OR 5 OR 6 OR 7 OR 8 OR 9 |
| 11 | exp medical records systems, computerized/ |
| 12 | "medical records system*".mp. |
| 13 | "patient administration system*".mp. |
| 14 | Medical Informatics/ or Medical Informatics Computing/ or Informatics/ or Medical Informatics Applications/ |
| 15 | "medical informatics".mp. |
| 16 | ("patient data" or "administrati* data" or "medical data" or "surgical data" or "practice data" or "performance data").mp. |
| 17 | ("clinical indicator*" or "quality indicator*" or "performance indicator*").mp. |
| 18 | 11 OR 12 OR 13 OR 14 OR 15 OR 16 OR 17 |
| 19 | user-computer interface/ |
| 20 | "user-computer interface*".mp. |
| 21 | data visualization/ |
| 22 | visuali#ation*.mp. |
| 23 | interface*.mp. |
| 24 | dashboard*.mp. |
| 25 | "web-based application*".mp. |
| 26 | 19 OR 20 OR 21 OR 22 OR 23 OR 24 OR 24 |
| 27 | 10 AND 18 AND 26 |
| 28 | LIMIT 27 TO (english language and yr=”2010 – 2020”) |
