# Supplementary material for: State-of-the-art Dashboards on Clinical Indicator Data to Support Reflection on Practice: Scoping Review
Source: JMIR Med Inform. 2022 Feb 14;10(2):e32695. doi: 10.2196/32695 (PMC8887640; doi:10.2196/32695)
Supplement: Multimedia Appendix 2 [file medinform_v10i2e32695_app2.docx]

## Multimedia Appendix 2

Summary of characteristics of studies included in the scoping review

| **Research design** | **First author (Year) [Ref]** | **Abridged title** | **Publication** | **Sample size** | **Evaluation methods** |
| --- | --- | --- | --- | --- | --- |
| Qualitative |  |  |  |  |  |
|  | Patel (2019) [25] | Development and implementation of maternity dashboard … | *Oman Medical Journal* | No details. | Prospective descriptive cross-sectional study |
|  | Leahy (2017) [32] | Making the pediatric perioperative surgical home … | *Current Opinion in Anesthesiology* | No details. | Retrospective case study |
| Mixed methods |  |  |  |  |  |
|  | Janssen (2020) [33] | Development of an intranet-based lymphedema dashboard … | *Journal of Medical Internet Research* | 5 health professionals | Think-Aloud Study, Questionnaire (SEQ), Interview |
|  | Mulhall (2020) [35] | Enhancing quality care in Ontario long-term care … | *Journal of the American Medical Directors Association* | 317 primary care physicians | Before-and-after study, Survey |
|  | Khanna (2019) [28] | Practice transformation analytics dashboard … | *The Annals of Family Medicine* | 11 primary care physicians, 14 other specialists | Before-and-after study, Survey |
|  | Gude (2018) [36] | Health professionals’ perceptions about their clinical performance … | *Implementation Science* | 84 intensive care physicians | Before-and-after study, Survey |
|  | Brown (2018) [38] | Multi-method laboratory user evaluation of an actionable … | *Journal of Biomedical Informatics* | 7 primary care physicians | Direct observation, Usability screen and eye-tracking, Questionnaire (SUS), Interview |
|  | Schall (2017) [24] | Usability evaluation and implementation of a health information technology … | *Computers, Informatics, Nursing* | 3 physicians, 3 nurses | Think-Aloud study, Questionnaire (SUS, PSSUQ) |
|  | Schall (2015) [26] | Development and evaluation of a health information technology … | *Proceedings of the Human Factors and Ergonomics Society Annual Meeting* | 7 healthcare professionals | Heuristic evaluation, Questionnaire (SUS) |
|  | Ehrenfeld (2014) [31] | Automated near-real-time clinical performance … | *Anesthesiology* | 60 anesthesia residents | Before-and-after study, Survey |
| Quantitative |  |  |  |  |  |
|  | Laurent (2020) [23] | Development, implementation and preliminary evaluation of clinical … | *Journal of Clinical Monitoring and Computing* | 12 anesthetists, 4 residents, 4 nurses | Questionnaire (SUS) |
|  | Hester (2019) [27] | Timely data for targeted quality improvement … | *Applied Clinical Informatics* | 57 emergency medicine physicians | Before-and-after study |
|  | Patel (2018) [29] | Next-generation audit and feedback for inpatient quality … | *BMJ Quality & Safety* | 24 internal medicine teams (1 attending physician, 1 resident, 2 internal medicine interns) | Cluster Randomized Control Trial |
|  | Herzke (2018) [30] | A method for attributing patient-level metrics … | *Journal of Hospital Medicine* | 20 physicians | Before-and-after study |
|  | Stattin (2016) [37] | Dashboard report on performance on select quality … | *Scandinavian Journal of Urology* | No details. | Before-and-after study |
|  | Weiner (2015) [22] | Integrating strategic and operational decision making … | *Journal of Healthcare Management* | No details. | Before-and-after study |
|  | Clark (2013) [34] | Breaking the mould without the system … | *Australian Health Review* | No details. | Before-and-after study |
|  | Linder (2010) [21] | Electronic health record feedback to improve … | *The American Journal of Managed Care* | 378 physicians | Cluster Randomized Control Trial |

**References**:

21 - Linder JA, Schnipper JL, Tsurikova R, Yu DT, Volk LA, Melnikas AJ, Palchuk MB, Olsha-Yehiav M, Middleton B. Electronic Health Record Feedback to Improve Antibiotic Prescribing for Acute Respiratory Infections. Am J Manag CARE 2010;16:9.

22 - Weiner J, Balijepally V, Tanniru M. Integrating Strategic and Operational Decision Making Using Data-Driven Dashboards: The Case of St. Joseph Mercy Oakland Hospital: J Healthc Manag 2015 Sep;60(5):319–330. doi: 10.1097/00115514-201509000-00005

23 - Laurent G, Moussa MD, Cirenei C, Tavernier B, Marcilly R, Lamer A. Development, implementation and preliminary evaluation of clinical dashboards in a department of anesthesia. J Clin Monit Comput [Internet] 2020 May 16 [cited 2021 Feb 12]; doi: 10.1007/s10877-020-00522-x

24 - Schall MC, Cullen L, Pennathur P, Chen H, Burrell K, Matthews G. Usability Evaluation and Implementation of a Health Information Technology Dashboard of Evidence-Based Quality Indicators. CIN Comput Inform Nurs 2017 Jun;35(6):281–288. doi: 10.1097/CIN.0000000000000325

25 - Patel M, Rathi B, Department of Obstetrics and Gynecology, Nizwa Hospital, A’Dakhiliyah, Oman, Yarubi MA, Department of Obstetrics and Gynecology, Nizwa Hospital, A’Dakhiliyah, Oman. Development and Implementation of Maternity Dashboard in Regional Hospital for Quality Improvement at Ground Level: A Pilot Study. Oman Med J 2019 May 19;34(3):194–199. doi: 10.5001/omj.2019.38

26 - Schall MC, Chen H, Pennathur PR, Cullen L. Development and Evaluation of a Health Information Technology Dashboard of Quality Indicators. Proc Hum Factors Ergon Soc Annu Meet 2015 Sep;59(1):461–465. doi: 10.1177/1541931215591099

27 - Hester G, Lang T, Madsen L, Tambyraja R, Zenker P. Timely Data for Targeted Quality Improvement Interventions: Use of a Visual Analytics Dashboard for Bronchiolitis. Appl Clin Inform 2019 Jan;10(01):168–174. doi: 10.1055/s-0039-1679868

28 - Khanna N, Gritzer L, Klyushnenkova E, Montgomery R, Dark M, Shah S, Shaya F. Practice Transformation Analytics Dashboard for Clinician Engagement. Ann Fam Med 2019 Aug 12;17(Suppl 1):S73–S76. doi: 10.1370/afm.2382

29 - Patel S, Rajkomar A, Harrison JD, Prasad PA, Valencia V, Ranji SR, Mourad M. Next-generation audit and feedback for inpatient quality improvement using electronic health record data: a cluster randomised controlled trial. BMJ Qual Saf 2018 Sep;27(9):691–699. doi: 10.1136/bmjqs-2017-007393

30 - Herzke CA, Michtalik HJ, Durkin N, Finkelstein J, Deutschendorf A, Miller J, Leung C, Brotman DJ. A Method for Attributing Patient-Level Metrics to Rotating Providers in an Inpatient Setting. J Hosp Med 2018 Jul 1;13(7):470–475. doi: 10.12788/jhm.2897

31 - Ehrenfeld JM, McEvoy MD, Furman WR, Snyder D, Sandberg WS. Automated Near–Real-time Clinical Performance Feedback for Anesthesiology Residents. Anesthesiology 2014 Jan 1;120(1):172–184. doi: 10.1097/ALN.0000000000000071

32 - Leahy IC, Borczuk R, Ferrari LR. Making the pediatric perioperative surgical home come to life by leveraging existing health information technology. Curr Opin Anaesthesiol 2017 Jun;30(3):383–389. doi: 10.1097/ACO.0000000000000454

33 - Janssen A, Donnelly C, Kay J, Thiem P, Saavedra A, Pathmanathan N, Elder E, Dinh P, Kabir M, Jackson K, Harnett P, Shaw T. Developing an Intranet-Based Lymphedema Dashboard for Breast Cancer Multidisciplinary Teams: Design Research Study. J Med Internet Res 2020 Apr 21;22(4):e13188. doi: 10.2196/13188

34 - Clark KW, Whiting E, Rowland J, Thompson LE, Missenden I, Schellein G. Breaking the mould without breaking the system: the development and pilot of a clinical dashboard at The Prince Charles Hospital. Aust Health Rev 2013;37(3):304. doi: 10.1071/AH12018

35 - Mulhall CL, Lam JMC, Rich PS, Dobell LG, Greenberg A. Enhancing Quality Care in Ontario Long-Term Care Homes Through Audit and Feedback for Physicians. J Am Med Dir Assoc 2020 Mar;21(3):420–425. doi: 10.1016/j.jamda.2019.11.017

36 - Gude WT, Roos-Blom M-J, van der Veer SN, Dongelmans DA, de Jonge E, Francis JJ, Peek N, de Keizer NF. Health professionals’ perceptions about their clinical performance and the influence of audit and feedback on their intentions to improve practice: a theory-based study in Dutch intensive care units. Implement Sci 2018 Dec;13(1):33. doi: 10.1186/s13012-018-0727-8

37 - Stattin P, Sandin F, Sandbäck T, Damber J-E, Franck Lissbrant I, Robinson D, Bratt O, Lambe M. Dashboard report on performance on select quality indicators to cancer care providers. Scand J Urol 2016 Jan 2;50(1):21–28. doi: 10.3109/21681805.2015.1063083

38 - Brown B, Balatsoukas P, Williams R, Sperrin M, Buchan I. Multi-method laboratory user evaluation of an actionable clinical performance information system: Implications for usability and patient safety. J Biomed Inform 2018 Jan;77:62–80. doi: 10.1016/j.jbi.2017.11.008
