# Supplementary material for: State-of-the-art Dashboards on Clinical Indicator Data to Support Reflection on Practice: Scoping Review
Source: JMIR Med Inform. 2022 Feb 14;10(2):e32695. doi: 10.2196/32695 (PMC8887640; doi:10.2196/32695)
Supplement: Multimedia Appendix 3 [file medinform_v10i2e32695_app3.docx]

## Multimedia Appendix 3

Summary of results of studies included in the scoping review

| **First author (Year) [Ref]** | **Results** | **Design methods** | **Indicators** | **Visual elements** | **Data source(s)** | **Technology platform** |
| --- | --- | --- | --- | --- | --- | --- |
| Laurent (2020) [23] | Mean SUS score 82.6 (SD 11.5) | User-centred design | *Unit management dashboard:*  No. of procedures, Mortality, Length of stay (LOS)  *Quality dashboard (ventilator guidelines):*  The mean tidal volume by year and by sex, The mean tidal volume based on the ideal body weight by year and by sex, The proportion of patients receiving more than 8 ml/kg, based on ideal body weight, The proportion of patients with a mean PEEP outside the range 5-10 cmH2O, The mean PEEP by year and by sex, The proportion of patients with a PP_lat_ over 30 cmH2O, The mean PP_lat_ by year and by sex.^a^ | Table, Bar chart/histogram | Data Warehouse | Custom - HTML/CSS/JS, PHP, Oracle, Chart.js and D3.js |
| Janssen (2020) [33] | All participants completed all the tasks on the individual patient dashboard (n=5), all tasks received a median rating of either 1 (very easy) or 2 (easy).  All tasks on the cohort dashboard received a median rating of 1 or 2. One task received a rating of 3, this task was only attempted by three of the participants. One participant was able to complete the first task on the cohort dashboard. | Co-design | Lymphedema index (L-Dex), No. nodes resected, Nodal positivity, No. procedures, No. radiotherapy patient, Patients by BMI, No. ongoing patient, No. recovered patients | Bar chart/histogram | EMR/EHR | Custom - jQuery, Store.js and HighCharts |
| Mulhall (2020) [35] | 50% of GPs viewed the online report (n=400) with 90% signing up for email delivery.  Rated quality of report: good (45%) and very good (34%).   69% likely or very likely to implement one of the changes.  Participants who viewed at least 1 of their report (n=260) had an almost 2% reduction in antipsychotic prescribing. | Co-design, User-centred design | Antibiotic prescribing rate, Antibiotic prolonged treatment rate, Antipsychotic prescribing rate, Benzodiazepine prescribing rate, CNS-active medications rate | Line chart | Clinical data sets, EMR/EHR, Bioimpedance spectroscopy machine data | No details |
| Hester (2019) [27] | 35% of ED clinicians logged in (n=57).  Improvements in all ED balancing measures: higher ED discharge rate (70.7 vs. 72.8, *P* = .05), lower charges (ration 1:.86, *P* < .001), shorter LOS (2.9 vs. 2.6 hours, *P* = .001), and lower 7-day revisits rates (15.4 vs. 11.6, *P* < .001).   Inpatient charges decreased (ratio 1:1.14, *P* = .01) but LOS and readmissions remained stable.  Timing of improvements and low individual clinician use suggest that the dashboard did not directly impact outcomes. | No details | LOS, No. admissions, No. inpatient visits per month, Admitted from ED %, Median ED stay charges, Median inpatient stay charges | Table, Bar chart/histogram | No details | QlikView |
| Khanna (2019) [28] | 24 of 25 had previously reviewed the dashboard data. 18 of 25 had favorable response to the dashboard. 12 of 25 found dashboard data actionable. | No details | Readmissions | Table, Line chart | No details | No details |
| Patel (2019) [25] | Clear and transparent dissemination of data enhanced the decision-making process. Analysis of the recorded data provided a powerful way to project the difference between the actual performance and expected goals. | No details | Deliveries, Admissions, Outpatient appointment, Instrumental deliveries, Cesarean section rate  Induction of labor, Midwife/patient ratio, Supervisor/midwife ratio, Eclampsia, Intensive care unit admission, Severe postpartum hemorrhage, Third-degree perineal tear, Shoulder dystocia, Hematomas, Postpartum hysterectomy, Other (near miss/mortality)  Low five-minute Apgar score (<7), Perinatal asphyxia, Meconium aspiration syndrome, Stillbirth, Stillbirth with diabetes, Early neonatal death. Patient complaints. | Table | No details | MS Excel |
| Patel (2018) [29] | Composite discharge matrix DMI: 79.3% completion in the intervention group (n=537) vs. 63.2% in the control group (n=516, *P* < .0001).  During the intervention period, the dashboard was accessed 104 times by 40 users in Feb, 77 times by 33 users in Mar, and 55 times by 30 users in Apr. During the washout period, 48 times by 20 users in May, 48 times by 24 users in Jun.  No significant difference in performance between the 'intensive' and 'usual' feedback groups. | No details | Discharge Mix Index (DMI), Home Med Rec Done, DC Summary in 24hrs, High quality after-visit summary (AVS) | Bar chart/histogram, Line chart, Pie chart | EMR/EHR | QlikView |
| Gude (2018) [36] | In 53.8% of cases (n=288), intensive care specialists overestimated their clinical performance, in 13.5% they underestimated.  Participants overestimated peer performance, and set targets 20.3% higher than the top performance benchmark.  In 68.4% of cases (n=197), intentions to improve practice were consistent with actual gaps in performance (without feedback), it increased to 79.9% after receiving feedback.  In 56.3% cases (n=40), participants still wanted to improve aspects they were already top performers and in 8.3%, they lacked improvement intentions as they did not consider indicators important. | No details | Performing pain measurements, Acceptable pain score, Repeating pain measurements with unacceptable score within 1 hr, Unacceptable pain scores normalised within 1 hr | Table, Bar chart/histogram, Line chart | No details | No details |
| Brown (2018) [38] | Efficiency: Task 2 had the highest median completion time overall (4.5 mins), Task 1 spent the longest time across all tasks (11.7 mins).  Median errors observed: 10 (range: 8-21, n=7)  Median task completion: 5 out of 7 (range: 4-7)  Usability heuristics were violated: 6 out of 38 (16%)  Median SUS score 73 (58-88). | No details | No. atrial fibrillation (AF) diagnosis over time, No. anticoagulation (A/C) under/overtreated, No. stroke risk patients, No. pulse rate patients, No. INR patients, No. patients excluded from AF standards | Table, Line chart | EMR/EHR, Patient Admin System | Custom - HTML, JS, CSS, Bootstrap, C3, jQuery |
| Herzke (2018) [30] | Provider performance above or below the median was in agreement 56-75% of the time (n=20).  The median difference between the methods was 13 to 22 percentile points for the various metrics. | No details | LOS Index, Readmit Index, % Discharged/Day, Discharges before 3pm, Patient satisfaction | Radar/spider chart | EMR/EHR, Patient Admin System | SAS, Web-based |
| Schall (2017) [24] | SUS mean 87.5 (SD 9.6) PSSUQ mean 1.7 (SD .5). System usability 1.5 (SD .4). Information quality 1.8 (SD .8). Interface quality: 1.8 (SD .8). | Focus groups | Pain rating, Fall risk, Pressure ulcer risk, Delirium risk, Barthel Index/Functional status, Restraint in use, Urinary catheter days, Central-line days, LACE score (readmission risk), Readmit (days since last readmission), Actual LOS, Expected LOS | Table | No details | No details |
| Leahy (2017) [32] | Dashboard format may improve clinicians’ ability to optimize health outcomes and contain healthcare costs. | Process mapping, Proof of concept pilot | No. patients, LOS, Max/avg cost, % readmissions, Day to readmissions | Bar chart/histogram, Line chart | EMR/EHR, patient 360 technology, Data Warehouse | No details |
| Stattin (2016) [37] | Proportion of patients reported in a timely fashion to registry increased from 26% in 2011 to 40% in 2013 (*P* < .001).  The use of active surveillance for men with very low-risk prostate cancer increased from 63% to 86% (*P* <.001).  In 2013 the overall median time from receipt of referral to the first visit to a specialist clinic was 35 days (IQR 21-58).  From prostate biopsy to the date when the patient received information on his cancer diagnosis was 29 days (IQR 21-40). | User-centred design, iterative process | Reported to NPCR, Navigator nurse, Waiting time to first visit, Waiting time to cancer diagnosis, Bone imagining (high risk cancer), Active surveillance (very low risk cancer), Multidisciplinary team meeting (high risk cancer), Curative treatment (localized high risk cancer), Nerve sparing intention, Negative margins | Bar chart/histogram, Line chart, Scatter plot | National Prostate Cancer Register | Custom - R, HighCharts.js |
| Weiner (2015) [22] | Acute patients (who may require admission) LOS (monthly) dropped by 54 minutes.  Lower-acuity patient (outpatients) LOS (monthly) dropped nearly an hour.  Number of patients in the ED who left without being seen fell from 165/month to 10/month. | Proof of concept pilot | *Emergency dashboard:*  ED visits, MTD ED discharges, Monthly LOS, Previous day treatment/release LOS, ED left w/o being seen MTD count, IP Order to placement TAT, Daily % of visits less than target  *Radiology dashboard:*  Previous day ED CT AT, Previous ED X-Ray TAT, Monthly test start to final dictate TAT trend, CT/MRI budgets | Table, Bar chart/histogram, Line chart, Meter/gauge | No details | No details |
| Schall (2015) [26] | 20 suggestions for change from heuristic evaluation. Five changes were proposed by 40% or more of the evaluators.   Mean SUS score of 83 (SD 7.6) | Focus groups, iterative design process | LOS, No. readmissions, Pain acceptable, Fall risk, Pressure ulcer risk, Barthel index, Delirium index, Restraint in use, Central line days, Urinary catheter days, Foley day | Table | No details | MS Excel |
| Ehrenfeld (2014) [31] | 91% responded they would like to receive systematic review of practice performance data every 1-4 weeks (n=48).  90% of residents responded that they could improve in at least one and often multiple areas (n=43).   10% believed that they were compliant in all of the six areas listed (n=5).  All respondents, except one, noted that would like to receive this feedback in some electronic form e.g. email, website, smartphone. | No details | Antibiotic administration, Central line, Glucose monitoring, Pain control, Temperature | Table, Line chart | Data Warehouse | Tableau |
| Clark (2013) [34] | Indicator performance improved an mean of 21.2% across the five indicators (range 8-38).  Est. date of discharge record with 12 hr of patient admission: 82% to 90%.  Patient management plan documented and communicated to patient within 24 hrs: 78% to 100%.  Criteria for discharge parameters documented and developed by MDT within 24 hrs of admission: 76% to 92%.  Discharge plan communicated to patient 24 hr before discharge: 48% to 86%.  Pharmacy script written 24 hr before patient discharge: 62% and 84%. | Stakeholder workshops | In ED waiting for EMU bed, Patients waiting transfer to sub-acute, Still waiting discharge criteria, Still waiting EDD, Still waiting notification of EDD, EDD tomorrow with no script in eDS, EDS outstanding summarises (pre-ward), Consultant workload, Admissions and discharges starting Tues 6am, Bed availability, Actual/projected admissions and discharges, Hospital in the home admissions | Bar chart/histogram, Line chart | No details | Custom - .NET, ASP.NET, SQL Server |
| Linder (2010) [21] | In the intent-to-intervene, there was no difference between intervention and control practices in antibiotic prescribing for all acute respiratory infection (ARI) visits.  Between practices, ARI dashboard use ranged from 0% (0 of 9 clinicians) to 67% (4 of 6 clinicians). The mean within-practice clinician use was 30% (SD 20%).  Within the intervention practices, 28% of clinicians used the dashboard at least once (n=72), these clinicians has lower overall ARI prescribing rates (42% vs 50%). | Proof of concept pilot | % acute respiratory infections (ARI) visits with antibiotics, % ARI visits with antibiotics by diagnosis, % broad spectrum prescribed in ARI visits with antibiotics, % ARI visits by level of service | Bar chart/histogram | Data Warehouse | Crystal Reports XI, ASP.NET |

^a^ Refer to Laurent (2020) for complete list of 39 different indicators across 17 dashboard themes.

**References**:

21 - Linder JA, Schnipper JL, Tsurikova R, Yu DT, Volk LA, Melnikas AJ, Palchuk MB, Olsha-Yehiav M, Middleton B. Electronic Health Record Feedback to Improve Antibiotic Prescribing for Acute Respiratory Infections. Am J Manag CARE 2010;16:9.

22 - Weiner J, Balijepally V, Tanniru M. Integrating Strategic and Operational Decision Making Using Data-Driven Dashboards: The Case of St. Joseph Mercy Oakland Hospital: J Healthc Manag 2015 Sep;60(5):319–330. doi: 10.1097/00115514-201509000-00005

23 - Laurent G, Moussa MD, Cirenei C, Tavernier B, Marcilly R, Lamer A. Development, implementation and preliminary evaluation of clinical dashboards in a department of anesthesia. J Clin Monit Comput [Internet] 2020 May 16 [cited 2021 Feb 12]; doi: 10.1007/s10877-020-00522-x

24 - Schall MC, Cullen L, Pennathur P, Chen H, Burrell K, Matthews G. Usability Evaluation and Implementation of a Health Information Technology Dashboard of Evidence-Based Quality Indicators. CIN Comput Inform Nurs 2017 Jun;35(6):281–288. doi: 10.1097/CIN.0000000000000325

25 - Patel M, Rathi B, Department of Obstetrics and Gynecology, Nizwa Hospital, A’Dakhiliyah, Oman, Yarubi MA, Department of Obstetrics and Gynecology, Nizwa Hospital, A’Dakhiliyah, Oman. Development and Implementation of Maternity Dashboard in Regional Hospital for Quality Improvement at Ground Level: A Pilot Study. Oman Med J 2019 May 19;34(3):194–199. doi: 10.5001/omj.2019.38

26 - Schall MC, Chen H, Pennathur PR, Cullen L. Development and Evaluation of a Health Information Technology Dashboard of Quality Indicators. Proc Hum Factors Ergon Soc Annu Meet 2015 Sep;59(1):461–465. doi: 10.1177/1541931215591099

27 - Hester G, Lang T, Madsen L, Tambyraja R, Zenker P. Timely Data for Targeted Quality Improvement Interventions: Use of a Visual Analytics Dashboard for Bronchiolitis. Appl Clin Inform 2019 Jan;10(01):168–174. doi: 10.1055/s-0039-1679868

28 - Khanna N, Gritzer L, Klyushnenkova E, Montgomery R, Dark M, Shah S, Shaya F. Practice Transformation Analytics Dashboard for Clinician Engagement. Ann Fam Med 2019 Aug 12;17(Suppl 1):S73–S76. doi: 10.1370/afm.2382

29 - Patel S, Rajkomar A, Harrison JD, Prasad PA, Valencia V, Ranji SR, Mourad M. Next-generation audit and feedback for inpatient quality improvement using electronic health record data: a cluster randomised controlled trial. BMJ Qual Saf 2018 Sep;27(9):691–699. doi: 10.1136/bmjqs-2017-007393

30 - Herzke CA, Michtalik HJ, Durkin N, Finkelstein J, Deutschendorf A, Miller J, Leung C, Brotman DJ. A Method for Attributing Patient-Level Metrics to Rotating Providers in an Inpatient Setting. J Hosp Med 2018 Jul 1;13(7):470–475. doi: 10.12788/jhm.2897

31 - Ehrenfeld JM, McEvoy MD, Furman WR, Snyder D, Sandberg WS. Automated Near–Real-time Clinical Performance Feedback for Anesthesiology Residents. Anesthesiology 2014 Jan 1;120(1):172–184. doi: 10.1097/ALN.0000000000000071

32 - Leahy IC, Borczuk R, Ferrari LR. Making the pediatric perioperative surgical home come to life by leveraging existing health information technology. Curr Opin Anaesthesiol 2017 Jun;30(3):383–389. doi: 10.1097/ACO.0000000000000454

33 - Janssen A, Donnelly C, Kay J, Thiem P, Saavedra A, Pathmanathan N, Elder E, Dinh P, Kabir M, Jackson K, Harnett P, Shaw T. Developing an Intranet-Based Lymphedema Dashboard for Breast Cancer Multidisciplinary Teams: Design Research Study. J Med Internet Res 2020 Apr 21;22(4):e13188. doi: 10.2196/13188

34 - Clark KW, Whiting E, Rowland J, Thompson LE, Missenden I, Schellein G. Breaking the mould without breaking the system: the development and pilot of a clinical dashboard at The Prince Charles Hospital. Aust Health Rev 2013;37(3):304. doi: 10.1071/AH12018

35 - Mulhall CL, Lam JMC, Rich PS, Dobell LG, Greenberg A. Enhancing Quality Care in Ontario Long-Term Care Homes Through Audit and Feedback for Physicians. J Am Med Dir Assoc 2020 Mar;21(3):420–425. doi: 10.1016/j.jamda.2019.11.017

36 - Gude WT, Roos-Blom M-J, van der Veer SN, Dongelmans DA, de Jonge E, Francis JJ, Peek N, de Keizer NF. Health professionals’ perceptions about their clinical performance and the influence of audit and feedback on their intentions to improve practice: a theory-based study in Dutch intensive care units. Implement Sci 2018 Dec;13(1):33. doi: 10.1186/s13012-018-0727-8

37 - Stattin P, Sandin F, Sandbäck T, Damber J-E, Franck Lissbrant I, Robinson D, Bratt O, Lambe M. Dashboard report on performance on select quality indicators to cancer care providers. Scand J Urol 2016 Jan 2;50(1):21–28. doi: 10.3109/21681805.2015.1063083

38 - Brown B, Balatsoukas P, Williams R, Sperrin M, Buchan I. Multi-method laboratory user evaluation of an actionable clinical performance information system: Implications for usability and patient safety. J Biomed Inform 2018 Jan;77:62–80. doi: 10.1016/j.jbi.2017.11.008
